# Diaphragmatic paralysis: a rare consequence of dengue fever

**DOI:** 10.1186/1471-2334-12-46

**Published:** 2012-02-22

**Authors:** Eranda C Ratnayake, Chrishan Shivanthan, Bandula C Wijesiriwardena

**Affiliations:** 1 Department of medicine (ward 45), the National hospital of Sri Lanka, Regent Street, Colombo 00800, Sri Lanka; 2 National Hospital of Sri Lanka, Regent Street, Colombo, Sri Lanka

**Keywords:** Dengue fever, Diaphragmatic paralysis, Phrenic neuropathy

## Abstract

**Background:**

Dengue is considered one of the most common mosquito borne illnesses in the world. Although its clinical course is usually uneventful, complications have rarely been known to arise. These include neurological manifestations such as neuropathies.

**Case presentation:**

We report a middle aged patient from urban Sri Lanka who developed diaphragmatic paralysis secondary to phrenic neuropathy a month after recovering from dengue fever. He was managed conservatively and made a full recovery subsequently.

**Conclusion:**

Isolated phrenic nerve palsy causing diaphragmatic paralysis should be considered a recognized complication of Dengue fever. A patient usually gains full recovery with conservative management.

## Background

Dengue virus infections are known to manifest in three main forms: - Dengue with or without warning signs and severe Dengue fever [1]. All three presentations of the disease usually recover uneventfully if accepted protocols are adhered to [2]. Uncommon manifestations of dengue fever, including neurological sequelae such as mononeuropathy, encephalopathy, transverse myelitis, polyradiculopathy and Guillain-Barre syndrome [3-5] have also been recognized in the past.

## Case presentation

**Figure 1:**
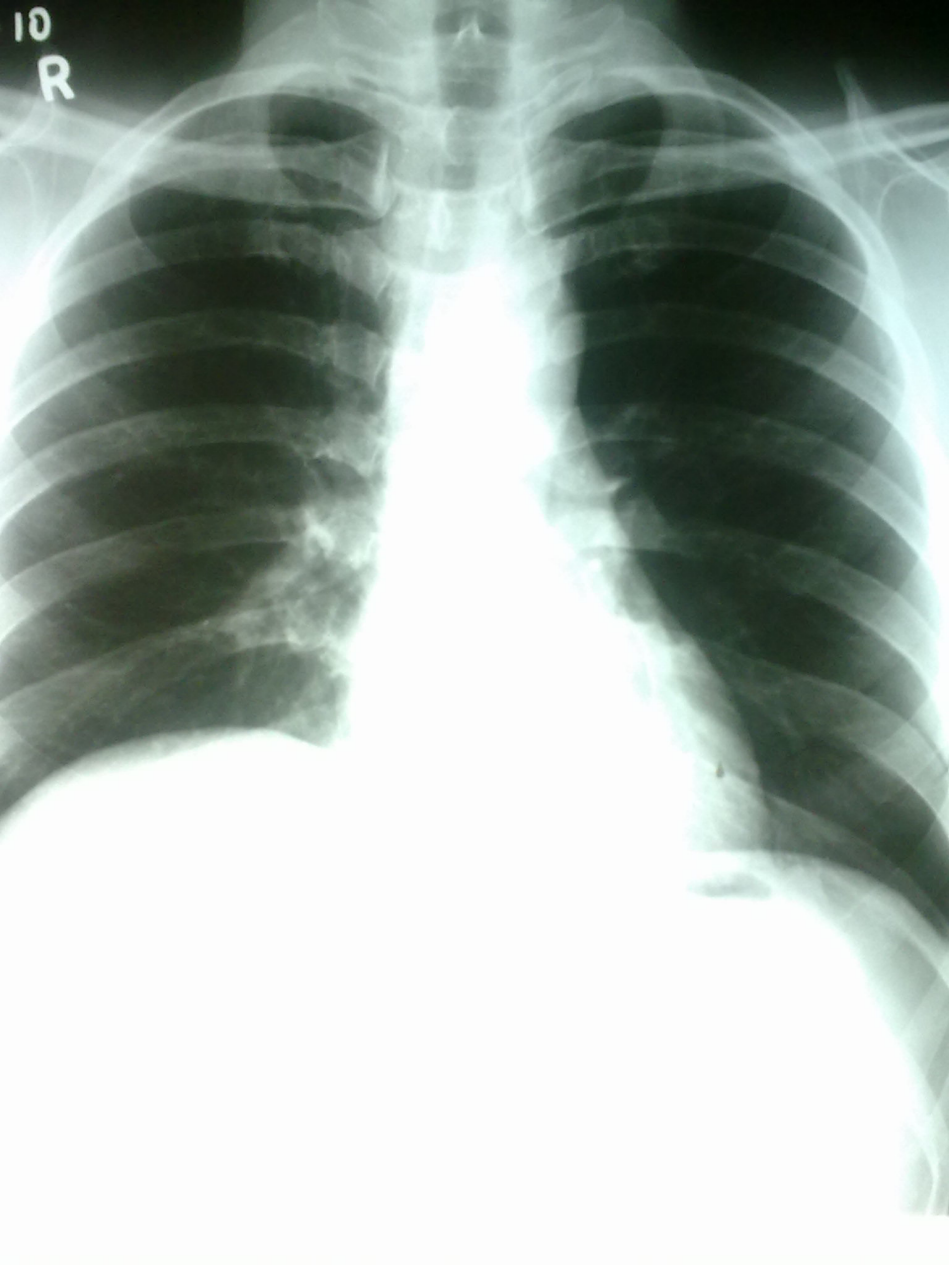
**Chest X-Ray on admission**.

A middle aged businessman from Colombo, Sri Lanka was admitted with a four day history of high fever, retro-orbital pain, athralgia and myalgia. He had no cough on admission and did not complain of breathlessness. He denied any bleeding manifestations including passing black stools. He also denied any recent travel to Malaria endemic regions in the country but had recently traveled to China on a business visit. He had no previous co-morbidities of significance. His physical examination was unremarkable on admission apart from a body temperature of 38.4°Celsius. His full blood count revealed a total white blood cell count of 5000/mm^3 ^with normal differentials, hemoglobin 17.4 g/dl with evidence of hemoconcentration (PCV 52%) and thrombocytopenia with a platelet count of 60,000/mm^3^. The platelet count dropped to a low of 20,000/mm^3 ^before recovery but the patient did not develop any significant bleeding manifestations. Chest X-ray on admission was normal (Figure 1).

Dengue fever was confirmed with a positive dengue polymerase chain reaction (PCR) result and positive Dengue IgM antibodies. He was managed with intravenous fluids according to national guidelines [2] and was discharged from hospital following a 48 h afebrile period, good appetite and adequate platelet rise. Two days prior to discharge he developed a persistent dry cough, without fever. A repeat chest x-ray was performed but did not reveal any abnormalities and he was treated for acute bronchitis with bronchodilators and cough suppressants.

**Figure 2:**
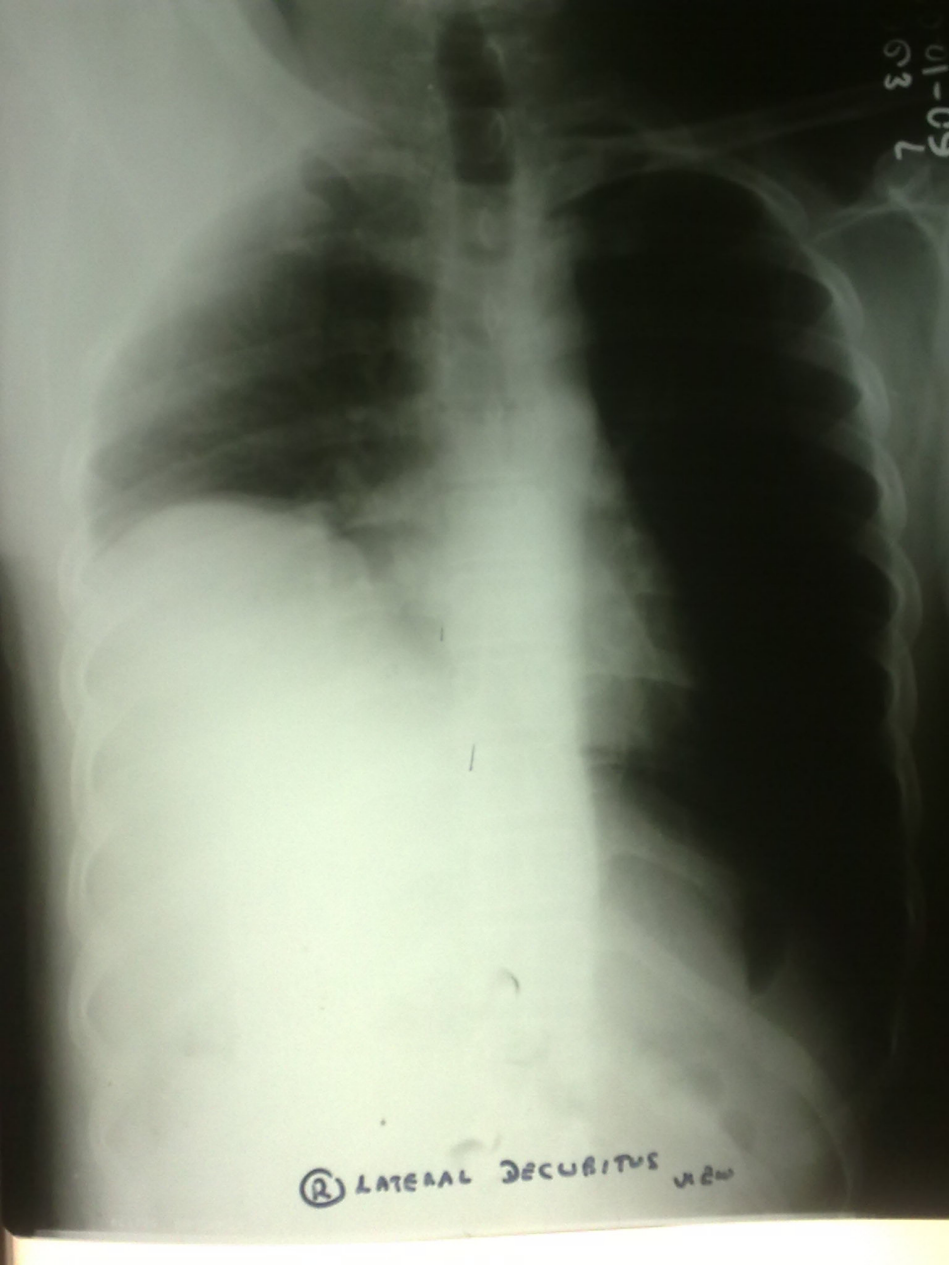
**Chest X-Ray one month after initial discharge**.

One month after discharge the patient was seen again, with complaints of a dry cough and breathlessness made worse with exertion (New York Heart Association Class III). The cough, which was present on initial discharge, had persisted but infrequently only to be continuous three days prior to the second admission. Orthopnoea was present but he did not complain of paroxysmal nocturnal dyspnoea. He had no wheezing, chest pain or palpitations. Physical examination at this point revealed normal vital parameters with decreased breath sounds in the right lung base with a stony dull percussion note. A chest x-ray was performed subsequently and showed an elevated right hemi-diaphragm (Figure 2).

**Figure 3:**
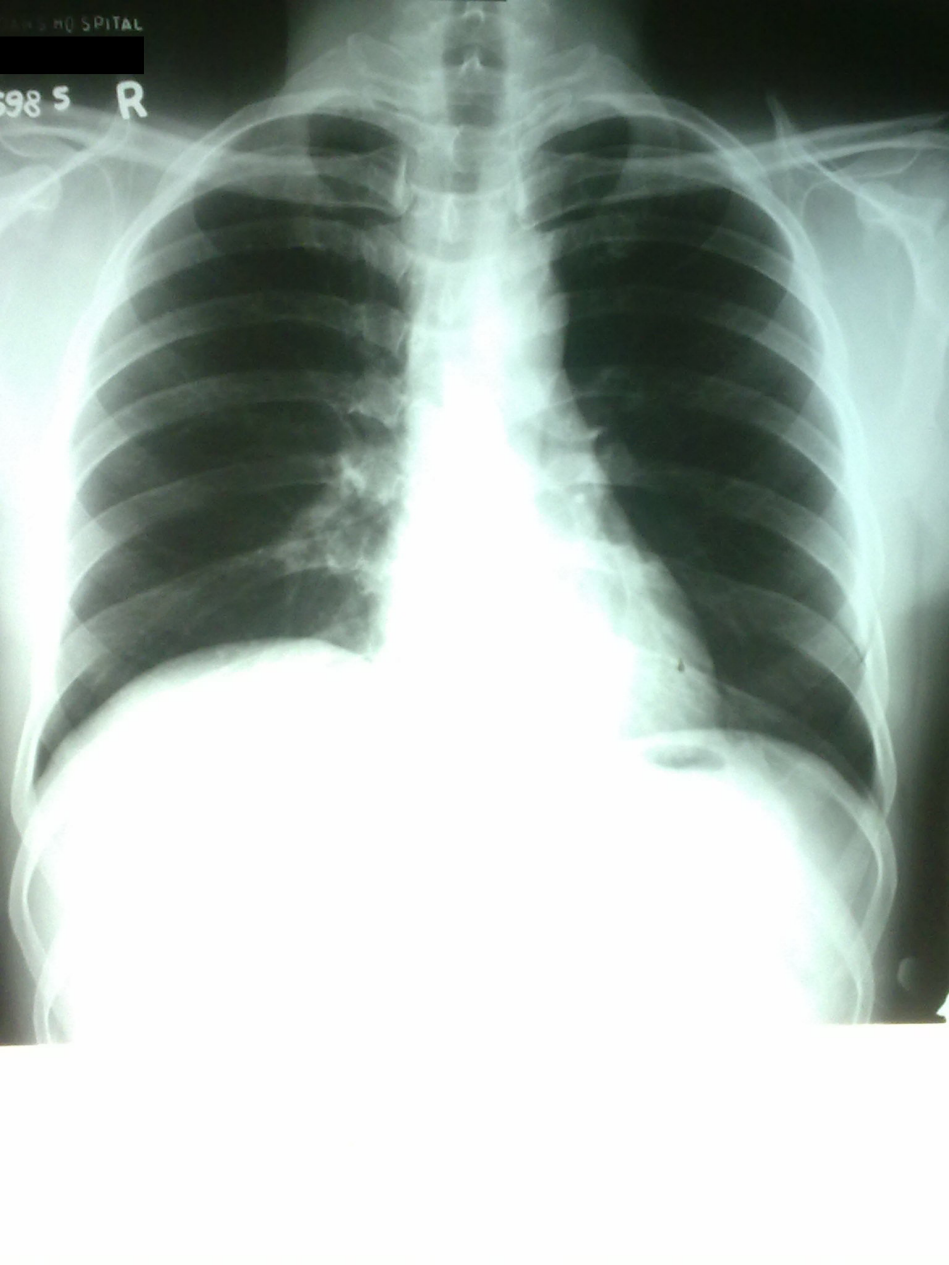
**Chest X-Ray two weeks after second discharge**.

Ultrasound scan chest did not reveal any pleural effusion but noted reduced diaphragmatic movements with respiration on the affected side. Contrast enhanced computed tomography (CECT) of the thorax did not reveal any lung parenchymal or mediastinal abnormalities. His lung function test revealed a sitting forced vital capacity of 2.11 L and supine 1.4 L (difference of 34%). He subsequently underwent nerve conduction studies of the phrenic nerves which revealed a decreased conduction amplitude on the right side suggestive of a demyelinating neuropathy, but was not suggestive of Guillain-Barre syndrome. There was no evidence of a neuromuscular junction disorder as suggested by normal electromyography. He was managed expectantly and spontaneously recovered within a few days of hospitalization and was discharged home. Repeat chest x-ray two weeks following discharge revealed that the hemidiaphragm was returning to normal position (Figure 3).

## Discussion

Dengue infection is the most common arboviral disease in the tropics and especially in Sri Lanka, where the season usually peaks in June-July each year. Although the clinical spectrum of the disease is well recognized, rare complications of the infection are encountered. Unusual neurological manifestations of the disease such as Encephalopathy and Guillain-Barre syndrome are reported more commonly than others [4]. Diaphragmatic paralysis due to phrenic nerve involvement has only been reported in two previous instances [6,7], both from the tropics. Our report introduces the third such patient with this very rare complication.

Our patient had confirmed dengue viral infection which was managed without complications in the ward. He was subsequently found to have a right sided phrenic nerve palsy a month later as confirmed by nerve conduction study and lung function tests. A myopathy or a neuromuscular junction pathology were excluded by normal electromyography. Phrenic nerve compression by mass lesions were excluded by a normal CECT. The presence of f waves on nerve conduction study made Guillain-Barre syndrome an unlikely cause although there was evidence of demyelination.

Post viral phrenic neuropathy has previously been documented following Polio-virus infection (particularly as a post-polio syndrome) [8], Herpes-Zoster infection [9] and Human Immunodeficiency virus infection [10]. The pathophysiology behind the manifestation remains obscure due to paucity of literature but an immune mediated mechanism is suggested [4]. Post viral phrenic neuropathy usually runs a self-limiting course, as was seen in our patient but may run a fulminant course with severe respiratory failure requiring mechanical ventilation [9].

## Conclusions

With dengue fever reaching and exceeding epidemic proportions in countries like Sri Lanka, more unusual complications of this very common disease are to be expected. In such light isolated phrenic nerve palsy causing diaphragmatic paralysis should be considered a recognized complication of this usually self limiting disease.

## Consent

Written informed consent was obtained from the patient for publication of this case report including all images. A copy of the written consent is available for review by the Editor-in-Chief of BMC Infectious Diseases.

## Competing interests

The authors declare that they have no competing interests.

## Authors' contributions

REC carried out the literature search and drafted the manuscript; WBC did the critical revision for important intellectual content in the manuscript and given the final approval of the version to be published; SC helped substantially in literature search and drafting the manuscript. All authors read and approved the final manuscript.

## Pre-publication history

The pre-publication history for this paper can be accessed here:

http://www.biomedcentral.com/1471-2334/12/46/prepub
